# Preliminary Support for a Cognitive Remediation Intervention for Women During the Menopausal Transition: A Pilot Study

**DOI:** 10.3389/fgwh.2021.741539

**Published:** 2021-12-23

**Authors:** Elena C. Ballantyne, Jelena P. King, Sheryl M. Green

**Affiliations:** ^1^Department of Psychiatry and Behavioural Neurosciences, McMaster University, Hamilton, ON, Canada; ^2^St. Joseph's Healthcare Hamilton, Hamilton, ON, Canada

**Keywords:** menopause, cognition, women's health, neuropsychology, cognitive remediation, cognitive confidence

## Abstract

**Background:** Menopause is associated with physical and emotional symptoms, and subjective cognitive concerns that are generally not borne out on objective cognitive measures. This discrepancy suggests that a psychological rather than biological mechanism likely mediates the cognitive concerns of women in menopause. The current study assessed the feasibility and effectiveness of a cognitive remediation intervention with the goal of reducing subjective perceptions of cognitive difficulty during the menopause.

**Methods:** Twenty-seven menopausal women (M age = 53.74, SD = 4.14) completed a 5-week group-based intervention (with a post-group booster) consisting of 2-h weekly sessions. Participants completed pre- and post-intervention measures capturing subjective cognitive ability, mood, anxiety, stress, personality, and objective cognitive tests. The primary variable of interest was self-reported cognitive confidence measured by the Memory and Cognitive Confidence Scale (MACCS).

**Results:** All but one MACCS subscale significantly decreased over the course of treatment (with lower scores associated with higher confidence) and effect sizes ranged from small to large (d = −0.39 to −0.91) with gains maintained at 1-month follow-up. Interestingly, no change in objective cognitive test performance was observed, indicating increases in subjective cognitive confidence in the absence of objective cognitive improvement. There was no change in mood, anxiety, or stress scores. Two-level HLM analyses revealed that those with higher baseline neuroticism, as measured by the NEO Personality Inventory, had smaller decreases in post-group MACCS High Standards subscale relative to those with lower baseline neuroticism (*p* = 0.027, d = −0.45). Those with higher baseline depression scores on the Depression Anxiety Stress Scale (DASS-21) had a smaller decrease in post-intervention MACCS Total Score relative to those with lower depression ratings.

**Conclusion:** To our knowledge, this is the first feasibility study of its kind targeting perceptions of cognitive impairment during menopause. Although generally well-tolerated, recruitment and scheduling difficulties were flagged as challenges to engagement while a small sample size and lack of control group limit conclusions about efficacy. Providing current results could be replicated with enhanced methods, these results provide support that cognitive remediation is a feasible and credible treatment, and may improve quality of life for women in menopause.

**Clinical Trial Registration:** www.ClinicalTrials.gov, identifier: NCT03311880.

## Introduction

Menopause, or the absence of a menstrual period for at least 12 consecutive months, is a natural biological transition that all women experience caused by a gradual decline in the reproductive hormones oestrogen and progesterone. According to a longitudinal study from the United States of an ethnically diverse sample of women between the ages of 42–52, the median age of the final natural menstrual period was 52.54 years (1). Common symptoms associated with the menopausal transition include vasomotor symptoms (e.g., hot flashes, night sweats), poor sleep (2, 3), mood disruption including symptoms of depression and anxiety (4), and urogenital problems and sexual concerns (5–7). These symptoms can be very distressing and disruptive for some women, negatively impacting their quality of life and overall functioning (8). In addition to physical and emotional symptoms, subjective perceptions of cognitive difficulty have also been frequently reported during the menopausal transition. For example, in a study by Mitchell and Woods (9), women in early, middle, and late stages of the menopausal transition between the ages of 35 and 55 completed open-ended interviews covering topics such as health and well-being, psychosocial stressors, menstrual cycle changes, role strain, and attitudes toward menopause and ageing. They were also asked about cognition with questions such as “Have you noticed any changes in your memory over the last few years?” Of the 230 women interviewed, 62% subjectively reported difficulties recalling names/numbers, forgetting events and actions, and concentrating. Another study examining subjective perceptions of post-menopausal Asian women between the ages of 40–65 from nine ethnic groups (10) revealed that 80.1% of the overall 1,028-person sample reported memory problems and 62.3% were experiencing concentration difficulties, though reporting rates varied by ethnicity.

However, results from studies examining the relationship between subjective cognitive complaints and objective measures of cognition have been mixed. For instance, in a study of 65 middle aged women by Drogos et al. (11), subjective appraisals of memory performance were compared to performance on standardised cognitive tests. While findings revealed an association between subjective memory concerns and declines in verbal memory on objective cognitive measures, cognitive test performance was within normal limits. These findings suggest that some middle-aged-women appear to notice subtle changes in cognition that is not indicative of bona-fide cognitive impairment. Another study by Unkenstein et al. (12) failed to identify cognitive impairment across menopause stage groups but found perimenopausal women reported more frequent memory complaints and dissatisfaction with their memory compared to the other groups. In contrast, other studies have revealed varying degrees of impairment in immediate and delayed memory (13, 14), phonemic fluency (15), and processing speed (13, 16), while others have failed to replicate these findings (17–19). For example, in a British study of 1,261 53-year-old women, there was weak evidence of the impact of natural menopause on cognition, and a suggestion that premenopausal cognition may account for the association between menopause and processing speed (20). While these findings have been mixed, a meta-analysis of cross-sectional studies investigating the presence of objective cognitive impairment in women during the menopausal transition revealed small effect sizes of cognitive changes overall (15). Relatedly, research investigating the impact of hormone replacement therapy (HRT) on cognition have been mixed with some studies indicating a beneficial effect (18, 21) while other work suggests no effect or worsening cognition (22–24). According to the critical window hypothesis (25), HRT may only be beneficial for healthy women earlier in the menopause transition (i.e., before the age of 60) as opposed to their post-menopausal counterparts, which may explain some of the variability across studies. More generally, critiques of the literature as a whole which may explain the discrepancies across studies of cognition in menopause include small sample sizes, inadequate cognitive test batteries (e.g., too insensitive, not geared toward detecting dementia-related changes), lack of control or comparison groups, failing to control for factors which may influence cognition (e.g., pre-morbid baseline, onset of natural menopause), few longitudinal studies, and inconsistent methodologies (e.g., conflicting menopause staging criteria, lack of consistency in menopausal stage examined) (15, 26, 27). In these regards, the current state of the literature does not provide compelling evidence that the menopausal transition is consistently associated with objective cognitive impairment (28) despite frequently reported subjective concerns and so additional factors must be considered.

Interestingly, discrepancies between objective and subjective measures of cognitive functioning have been reported in other conditions such as insomnia (29), bipolar disorder (30), anxiety (31), and depression (32), where subjective cognitive complaints are associated with psychological symptom severity or perceived level of distress rather than actual cognitive impairment, *per se* (33). In a similar way, a psychological mechanism might mediate the subjective perception of cognitive difficulties and associated distress women report during the menopausal transition. For example, the role of emotional health, stress, culture, and beliefs and attitudes toward menopause has been identified as important factors contributing to women's experience of menopause (34–36). Relatedly, Brown et al. (37) propose that women's emotional and cognitive conceptualisation of menopause may vary depending on their particular stage of menopause which the authors attribute to psychological mechanisms such as improved coping and self-efficacy over time and affect forecasting theory. In a similar vein, Hunter and O'Dea (38) examined women's cognitive appraisal of menopause, examining the impact of psychological factors such as attribution of symptoms, perceived time-frame of symptoms, self-identity, consequences of symptoms, and perceptions of control and cure. These psychological factors were identified as important determinants of whether a woman's experience of the menopausal transition was positive, negative, or neutral. Although little attention has been paid to identifying the psychological mechanisms that predict greater subjective cognitive complaints in the menopausal transition (that might therefore also play a role in change during treatment), women who report higher levels of affective distress (i.e., depression, anxiety) during menopause also report greater subjective cognitive complaints, even in the absence of objective deficits (39, 40). Moreover, psychological factors that predict discrepancies in subjective and objective cognitive complaints in other populations (e.g., individuals with mood or anxiety disorders) may also be relevant here and may include perfectionistic beliefs about cognitive performance, low confidence in cognitive abilities (41) and negative beliefs about the effect of ageing on cognition.

Non-pharmacological approaches such as cognitive-behavioural therapy (CBT) and mindfulness-based stress reduction therapy (MBSR) have been shown to be effective with respect to attenuating distress and reducing the intensity of physical and emotional symptoms experienced by women during the menopausal transition (42–44). However, many of these studies contain similar methodological flaws as described above, thus limiting their conclusions (45). Additionally, these interventions have not been specifically designed to target or remediate self-reported cognitive difficulties, though they may indirectly improve cognition by reducing affective distress, which is known to disrupt frontal lobe functioning (46–49). Currently, there is no psychological intervention that directly targets/remediates cognitive difficulties and associated emotional distress that some women experience during the menopausal transition. However, some treatment recommendations of cognitive symptoms during the menopausal transition do exist such as psychoeducation about severity and anticipated duration of cognitive symptoms and possible contributing factors (e.g., depressed mood, sleep disruption, situational stressors), normalisation of subjective cognitive complaints, group-based discussions about personal, health and social issues during menopause, examination of attitudes toward menopause and provision of reassurance for overly pessimistic beliefs, interventions that reduce cardiovascular risk (e.g., exercise, smoking cessation), promotion of mental wellness (e.g., stress reduction techniques, psychotherapy, improving sleep), inclusion of compensatory strategies (e.g., mnemonic devices), and consideration of cognitive testing if functioning is impaired (34, 50, 51).

The importance of developing such interventions is underscored by research demonstrating that many women in the menopausal transition are concerned that perceived cognitive changes may signal the onset of dementia rather than reflect normal hormonal changes (9, 39). Fears of developing dementia in and of itself likely contribute to increased psychological distress and exacerbation of self-reported cognitive and functional impairment. Indeed, research has shown that negative expectations regarding one's performance can have negative effects on actual task performance (52–54) which is particularly concerning as many middle-aged women continue to contribute to the workforce and aspire to maintain their pre-menopausal level of cognitive performance. Additionally, it is important to offer non-pharmacological options for symptom management, particularly for women who are unable to receive HRT or prefer non-pharmacological approaches.

To address this clinical and research gap, we developed a brief intervention for menopausal women targeting self-reported cognitive difficulties. The current investigation is a feasibility study, as defined by Eldridge et al. (55), of a group-based cognitive remediation intervention for women in the menopausal transition. The primary objectives were to evaluate the feasibility and acceptability of this group to prepare for a possible future, larger-scale study and to evaluate whether the intervention (consisting of psychoeducation, cognitive compensatory strategies, and lifestyle modification) improves self-reported cognitive difficulties. Relative to pre-treatment, we anticipated that participants would experience an improvement in self-reported confidence and attenuated distress regarding subjective cognitive impairments with no accompanying improvement in cognitive ability on objective tests of cognitive function which was anticipated to fall within normal limits. This would support the notion that psychological rather than biological mechanisms mediate subjective perceptions of cognitive difficulties for women during the menopausal transition.

A secondary objective of the study was to identify factors or mechanisms that promoted or hindered clinical change within the current intervention to inform future treatment development (56–59). For example, psychological distress (depression, anxiety, stress) and pre-existing personality processes such as perfectionism and neuroticism have been implicated in maintaining symptomatology in other populations with cognitive concerns such as traumatic brain injury (60, 61) and may serve as a vulnerability for decreased resiliency after a perceived decline in cognition.

## Materials and Methods

### Ethics

This study received ethics approval from the Hamilton Integrated Research Ethics Board (HiREB) on November 20, 2018 (reference number 2017-2950-GRA). All participants provided written informed consent.

### Participants

**Figure 1:**
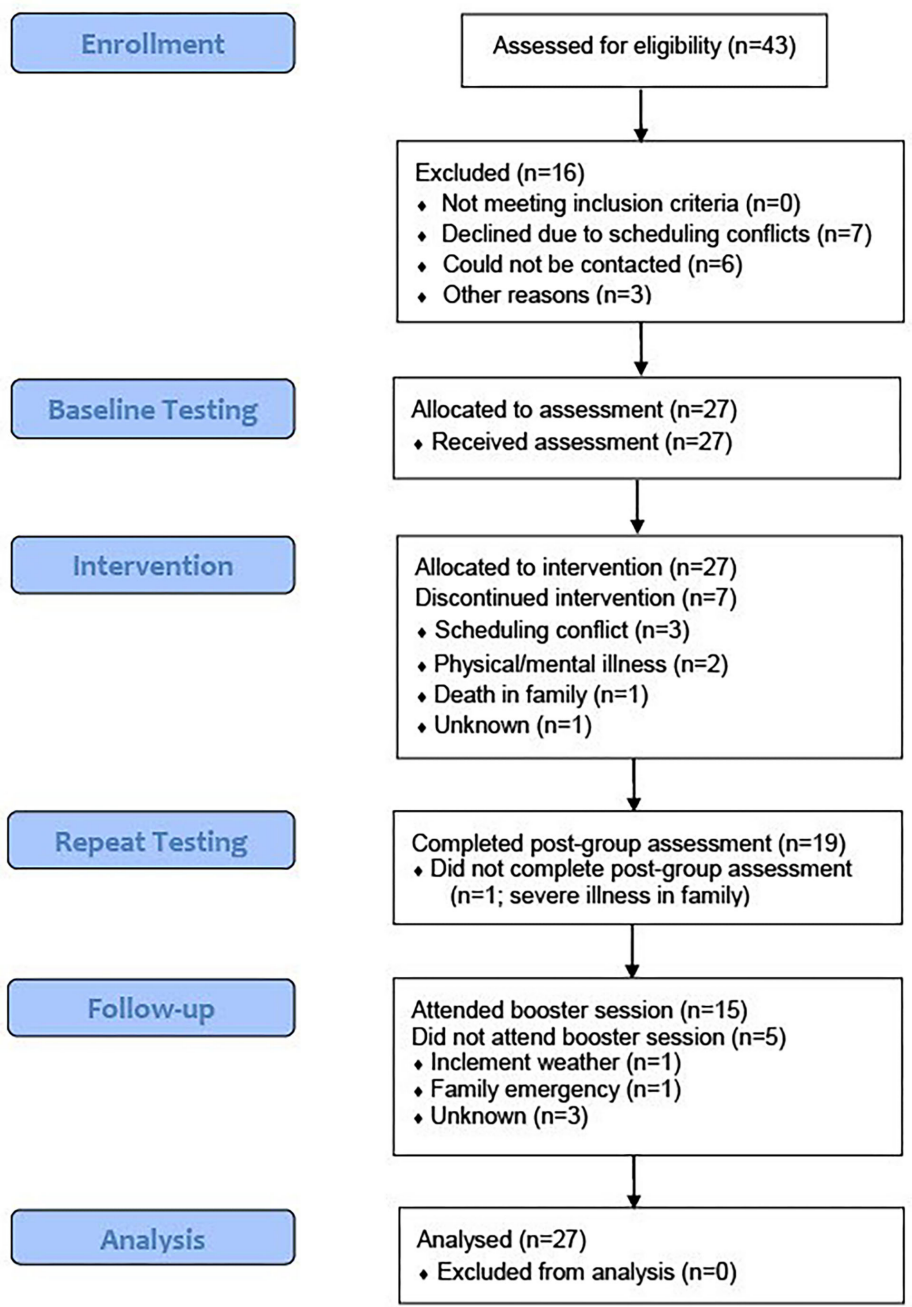
CONSORT flow diagram.

Target sample size (*n* = 40) was selected based on other similar cognitive remediation feasibility studies [e.g., (62–64)]. Participants (*n* = 27) were women who were either registered patients of St. Joseph's Healthcare Hamilton, referred to the group by a regulated health professional, or individuals recruited through the community *via* newspaper and community presentations. Eligibility criteria were as follows: 40–65 years of age; fluency in English, perimenopausal or postmenopausal or surgically induced menopause; any subjective cognitive complaints (new or worsening symptoms such as inattention, forgetfulness, or brain fog within the menopausal transition); experiencing at least one vasomotor symptom (hot flashes and/or night sweats). Additional eligibility criteria included: if taking medication, dose and type of medication were consistent for 3 months prior to baseline testing; no changes in dose or type of medication throughout the study. Exclusion criteria included: untreated and severe PTSD, mania, psychosis, substance abuse, suicidal ideation; medical conditions that would likely interfere with participation (e.g., dementia, severe traumatic brain injury, mild cognitive impairment). See Figure 1 for a CONSORT diagram of dropout or loss to follow-up.

### Procedure

This study employed a single-sample, pre-post study design combined with a repeated measures process design. Prior to beginning the intervention, participants completed paper-based demographic information forms and questionnaire batteries consisting of all the self-report measures listed below assessing perceived cognitive functioning, distress and interference from hot flashes, psychological distress, and personality. Each participant also underwent a comprehensive screening of objective cognitive ability where they completed neuropsychological tests assessing overall intellectual functioning, attention, memory, processing speed, and executive functioning ability. Process of change variables (degree of affective distress, confidence in cognitive abilities) were assessed at the outset of each session using two questionnaires administered at baseline (Depression Anxiety Stress Scales, Memory and Cognitive Confidence Scale). At the end of the group, participants completed an abbreviated self-report questionnaire and cognitive test battery using a subsection of tests administered at baseline. Since this was a feasibility study, we solicited feedback on the program using a post-intervention questionnaire which included 7-point Likert scales and open-ended questions regarding how helpful participants found the group, to what extent strategies were used in daily life, and suggestions for future groups at 1-month follow-up. Measures of cognitive confidence and affective distress were also repeated at a 1-month booster session to determine whether any additional change took place over time.

Cognitive Remediation Intervention: The Menopause and the Brain Group protocol consisted of five, weekly 2-h in-person sessions. A session-by-session description is provided in Table 1. The structure and style of the protocol was similar in style to other cognitive remediation interventions (65, 66) and incorporated recommended guidelines for non-pharmacological menopause-related interventions described above. The groups were delivered by two licenced clinical neuropsychologists. Each session consisted of didactics (e.g., basic brain anatomy, summary of literature regarding cognition in menopause, effects of attitude on menopause and cognition, dementia-related concerns), group discussion about the material, and goal setting. Cognitive compensatory strategies were incorporated into each session. For example, the “pay attention to stress” session introduced concepts such as reducing distractions (e.g., eliminating visual clutter, turning off cell phone/TV, avoid multi-tasking), pacing, and optimising scheduling (e.g., avoid back-to-back appointments, scheduling cognitively demanding tasks in the “freshest” part of the day). Given the potential role of medical factors in cognitive decline, relevant lifestyle strategies were also discussed in relation to the targeted area of cognition. For example, participants were informed about the relationship between stress and cognition, followed by brief guided imagery and deep breathing exercises. Each session was accompanied by a handout which summarised the psychoeducational information and relevant worksheets. A booster session was held 1-month after group completion to review content from the group, review home practise goals, and discuss maintaining gains.

**Table 1:** Description of menopause and the brain sessions.

**Session**	**Title**	**Content**
1	What is Menopause?	Psychoeducation about menopause stages and common symptoms, research findings about subjective and objective cognitive impairment during menopause, normal vs. pathological ageing, factors beyond menopause that impact cognition
2	Pay attention to stress	Description of attentional processes, common symptoms during menopause, psychoeducation about the impact of stress on cognition and the brain, in-session relaxation practise, review of compensatory strategies, goal setting
3	Remember to exercise	Description of memory processes, common memory slips during menopause, psychoeducation about benefits of exercise on cognition and physical health, review of compensatory strategies, goal setting
4	Plan your way to healthy eating	Description of executive functioning and common symptoms, SMART goals, impact of diet on cognition and physical health, review of compensatory strategies, goal setting
5	Think about calling a friend	Psychoeducation about cognitive and social engagement on brain health, goal setting
6	Booster session	Review of previous goals and skills, group discussion, plan for maintaining gains

### Measures

*The Memory and Cognitive Confidence Scale* (MACCS) (41). The primary outcome was participants' level of self-reported confidence in their cognition using the MACCS which is a self-report measure consisting of four subscales: beliefs about general memory abilities, confidence in decision-making abilities, confidence in one's ability to focus or concentrate, and high standards regarding one's cognitive performance. This scale has been found to have satisfactory internal consistency for each of the four subscales and the total scale.

*Brief Visuospatial Memory Test* (BVMT-R) (67). The BVMT-R is a measure of simple visual memory which includes immediate recall, delayed recall, and recognition trials.

*California Verbal Learning Test* (CVLT-II) (68). The CVLT-II is a measure of verbal learning and memory, and contains encoding, interference, recall, and recognition trials.

*Controlled Oral Word Association Test* (COWAT) (69). The COWAT was used to assess language functioning, specifically phonemic and semantic fluency.

*Depression, Anxiety, and Stress Scale* (DASS-21) (70) was used to assess psychological distress. Scores were multiplied by two to be comparable with the original DASS clinical cut-offs. The internal consistency and concurrent validity were in the acceptable to excellent range and were replicated from previous studies (71).

*The Hewitt-Flett Multidimensional Perfectionism Scale* (72) is a self-report measure of perfectionistic traits with respect to self, other, and socially oriented perfectionism. Internal consistencies range from 0.74 to 0.88 for the subscales.

*The Hot Flash Rating Scale* (HFRS) (73) assessed distress and interference associated with hot flashes and night sweats to quantify the presence of vasomotor symptoms. The two factors of frequency (hot flushes/night sweats) and problem ratings (distress, interference and perception of flushes as problematic) had high test-retest reliability, and the frequency ratings correlated highly with prospective daily monitoring.

*The Mini International Neuropsychiatric Interview for DSM 5* (MINI) (74) is a short, structured diagnostic interview for mental health conditions in the DSM-5. Kappa coefficient, sensitivity and specificity were good or very good for most diagnoses and interrater and test-retest reliability were good (75).

*Montgomery-Asberg Depression Rating Scale* (MADRS) (76) was administered to participants at the outset of the study to characterise the sample. Inter-rater reliability using the Structured Interview Guide for the Montgomery-Asberg Depression Rating Scale (SIGMA) was r = 0.93 (77).

*NEO Personality Inventory* (NEO-FFI) (78). Personality traits were measured by the NEO-FFI which is a 60-item self-reported personality inventory examining the Big 5 personality traits. Internal consistency among the NEO scales ranges from 0.56 to 0.81.

*Test of Premorbid Functioning* (TOPF) (79). Pre-morbid intellectual functioning was estimated using the TOPF which is a single word reading test.

*Trail Making Test, Part A* (80). Processing speed was measured by the Trails A portion of the Trail Making Test which involves speeded visual scanning and numerical sequencing.

*Wechsler Abbreviated Scale of Intelligence* (WASI-II) (81). Current intellectual functioning was measured using the WASI-II which is an abbreviated measure of intelligence.

*Wechsler Adult Intelligence Scale* (WAIS-IV) (82). Attention and working memory were assessed using the Digit Span subtest from the WAIS-IV. The Reliable Digit Span was also used as an embedded validity indicator (83).

*Wechsler Memory Scales* (WMS-III) (84). The Spatial Span subtest from the WMS-III was used to assess visual attention and working memory.

At the conclusion of the group, participants were asked to rate how helpful they found the group using a Likert scale from 0 to 6 (0 = not at all; 6 = extremely) and how often they used skills learned in the group using another Likert scale from 0 to 6 (0 = not at all; 6 = totally).

### Statistical Analysis

Primary outcome variables were analysed using one-level hierarchical linear modelling (HLM) (85) to assess changes in outcome variables over time. The data points included pre- and post-treatment assessments as well as session-by-session data (DASS and MACCS questionnaires), with the unit of time coded as 1 week. HLM was used given its ability to evaluate individual differences in trajectory of change over time, and because it can accommodate missing data at Level-1. We also conducted several two-level models to determine the impact of baseline variables on the trajectory of change in primary outcomes over the course of treatment. The Level-2 variables of interest, based on a priori hypothesis, were the HPS Self-Oriented Perfectionism subscale and the NEO Neuroticism *T* Score, and were performed on those subscales of the MACCS observed to be significant in the one-level analyses. Means of Level-2 variables were centered around the grand mean. Finally, for any main outcomes seen to be significant in the primary analysis, a follow-up *t*-test was conducted between the final session and the 1-month follow-up session to determine whether gains were maintained. Despite the pilot nature of the present study and the emphasis on effect size rather than statistical significance alone, all analyses were planned with the intent to reduce the total number of comparisons being made, reducing risk of Type I error as much as was feasible. Paired samples *t*-tests were also performed on pre-post measures of neuropsychological performance to rule out neurocognitive changes as potential covariates.

## Results

A total of 27 women were included in the study. In terms of attrition, of the seven participants who dropped out of the group, three participants dropped out due to scheduling conflicts, one participant withdrew from the study because of a death in the family while two participants withdrew due to a medical illness and psychiatric issues, respectively. One participant completed the baseline testing but did not attend any group sessions for unknown reasons. Seventy-four percent of participants (*n* = 20) completed the group with completion defined as missing two sessions or less. Twelve participants (44%) completed every session (and thus completed all survey and cognitive data) including the booster session 1 month after group completion. Six participants missed one session, two participants missed two sessions while an additional two participants missed three sessions. Reasons for missing sessions included illness, inclement weather, personal/family emergencies, and mental health issues. In three instances, the reason for the individual's absence was unknown. Baseline assessment data were collected for all 27 participants. In total, eight people did not complete post-group repeat testing.

All mean scores on intellectual and neuropsychological tests fell within the average or high average range compared to normative data sets. There were no significant differences observed between any variables of neuropsychological task performance between pre- and post-treatment (*p*s > 0.050). Scores from the self, other, and socially oriented subscales of the HPS fell within the average range compared to same-aged peers. With respect to the NEO, our sample had average mean scores in the domains of openness, agreeableness, and conscientiousness. Mean neuroticism was high average while mean extraversion was low average. Participant demographics are summarised in Table 2 and baseline and post-group cognitive and psychological characteristics can be found in Table 3.

**Table 2:** Sample demographics.

**Variable**	**Frequency**
Age, mean (SD)	53.74 (4.14)
Relationship status (% married)	59.3%
Employment status (% full-time)	48.1%
Family income status (% ≥$100,000)	44.4%
Education (% ≥Undergraduate Degree)	55.5%
Psychiatric diagnosis (% Yes)	70.4%
Generalised anxiety disorder	14.8%
Major depressive disorder, recurrent	11.1%
Major depressive episode, current	48.1%
Major depressive episode, past	77.8%
Obsessive-compulsive disorder	3.7%
Panic disorder	7.4%
Posttraumatic stress disorder	11.1%
Medication status (% yes)	40.6%
SSRI	25.9%
SNRI	14.8%
Atypical anti-psychotic	0.04%
Hormone replacement therapy	19%
Counselling/therapy status (% yes)	33.3%

*All but two participants made no changes to their medication regimen over the course of the study, aside from one person who discontinued Premarin and another who discontinued her anti-depressant*.

**Table 3:** Baseline and post-group cognitive and psychological sample characteristics.

	**Baseline**	**Post-group**
**Variable**	**Mean (SD)**	**Mean (SD)**
DASS-21^*^ Depression	14.15 (10.11)	10.78 (11.54)
DASS-21 Anxiety	7.56 (6.80)	4.22 (5.26)
DASS-21 Stress	16.37 (10.87)	15.00 (9.78)
HPS Self *Z*-score	−0.04 (1.21)	0.05 (1.29)
HPS Other *Z*-score	−0.19 (1.15)	−0.02 (0.90)
HPS Social *Z*-score	−0.06 (1.05)	0.18 (1.14)
TOPF Standard Score	106.07 (11.20)	–
WASI Vocab *T* Score	56.15 (6.64)	–
WASI Matrix Reasoning *T* Score	56.67 (10.11)	–
WASI FSIQ Standard Score	111.04 (13.10)	–
Trails A *T* Score	54.19 (10.90)	59.95 (11.45)
Trails B *T* Score	56.56 (11.30)	59.05 (10.89)
CVLT-II Trials1-5 *T*-Score	60.37 (10.80)	64.00 (9.96)
CVLT-II List B Z Score	0.41 (0.95)	0.47 (1.05)
CVLT-II Short-Delay Free Recall Z-Score	0.85 (1.07)	0.95 (1.04)
CVLT-II Short-Delay Cued Z-Score	0.67 (0.84)	0.82 (0.79)
CVLT-II Long-Delay Z-Score	0.74 (0.89)	0.60 (0.84)
CVLT-II Long-Delay Cued Z-Score	0.74 (0.76)	0.76 (0.77)
CVLT-II Discrimination Z-Score	0.78 (0.74)	0.84 (0.91)
BVMT-R Trials 1–3 Total *T*-Score	52.63 (11.01)	62 (6.81)
BVMT-R Delayed Recall *T*-Score	56.33 (0.24)	61 (4.02)
BVMT-R Recognition Raw	5.74 (0.53)	5.89 (0.32)
WAIS-IV Digit Span Forward Scaled Score	10.22 (3.09)	10.26 (2.18)
WAIS-IV Digit Span Backwards Scaled Score	10.89 (2.9)	11.79 (2.27)
WAIS-IV Digit Span Sequences Scaled Score	11.56 (2.87)	12.42 (1.98)
COWAT (FAS/CFL) Total *T*-Score	44.26 (13.44)	51.42 (5.53)
COWAT (Animals) Total *T*-Score	51.07 (10.57)	56.05 (8.87)
WMS-III Spatial Span Total Scaled Score	12.26 (2.5)	12.05 (2.27)

* *DASS-21 scores have been multiplied by 2 to be comparable with the original DASS clinical cut-offs*.

### One-Level HLM Analyses

Results from the one-level HLM analyses of primary outcomes can be found in Table 4. In terms of self-reported cognitive confidence, all subscales of the MACCS were seen to significantly decrease over the course of treatment, aside from the High Standards subscale, which was only approaching significance (*p* = 0.052). Effect sizes for all subscales ranged from small to large (d = −0.39 to −0.91). In terms of psychological functioning, none of the three DASS-21 subscales changed significantly from pre- to post-treatment, meaning they remained slightly elevated overall.

**Table 4:** One-Level hierarchical linear modelling assessing the change in primary outcome variables over time from baseline to 1-month follow-up.

**Effect**	**b**	**SE**	**t**	**df**	***p***	**d**
**MACCS total score**						
Initial MACCS (intercept)	94.52	3.09	30.60	26	<0.001	
MACCS over time (slope)	−2.90	0.62	−4.69	26	<0.001	0.90
**MACCS attention/concentration subscale**						
Initial MACCS AC (intercept)	14.46	0.72	20.13	26	<0.001	
MACCS AC over time (slope)	−0.37	0.13	−2.95	26	0.007	−0.57
**MACCS decision-making subscale**						
Initial MACCS DM (intercept)	16.75	0.89	18.84	26	<0.001	
MACCS DM over time (slope)	−0.62	0.14	−4.59	26	<0.001	−0.88
**MACCS general memory subscale**						
Initial MACCS GM (intercept)	46.89	1.48	31.73	26	<0.001	
MACCS GM over time (slope)	−1.59	0.34	−4.74	26	<0.001	−0.91
**MACCS high standards subscale**						
Initial MACCS HS (intercept)	13.36	0.86	15.49	26	<0.001	
MACCS HS over time (slope)	−0.22	0.11	−2.04	26	0.052	−0.39
**DASS-21 depression subscale**						
Initial DASS-D (intercept)	15.84	1.97	8.04	26	<0.001	
DASS-D over time (slope)	−0.27	0.28	−0.96	26	0.346	−0.18
**DASS-21 anxiety subscale**						
Initial DASS-A (intercept)	7.91	1.27	6.22	26	<0.001	
DASS-A over time (slope)	−0.15	0.17	−0.86	26	0.401	−0.17
**DASS-21 stress subscale**						
Initial DASS-S (intercept)	9.39	0.83	11.30	26	<0.001	
DASS-S over time (slope)	−0.04	0.17	−0.25	26	0.805	−0.05

*DASS-21, Depression Anxiety and Stress Scales, 21-item version; DASS-A, Anxiety Subscale; DASS-D, Depression Subscale; DASS-S, Stress Subscale; MACCS, Memory and Cognitive Confidence Scale; MACCS AC, Attention/Concentration Subscale; MACCS DM, Decision-Making Subscale; MACCS GM, General Memory Subscale; MACCS HS, High Standards Subscale*.

### Two-Level HLM Analyses

**Figure 2:**
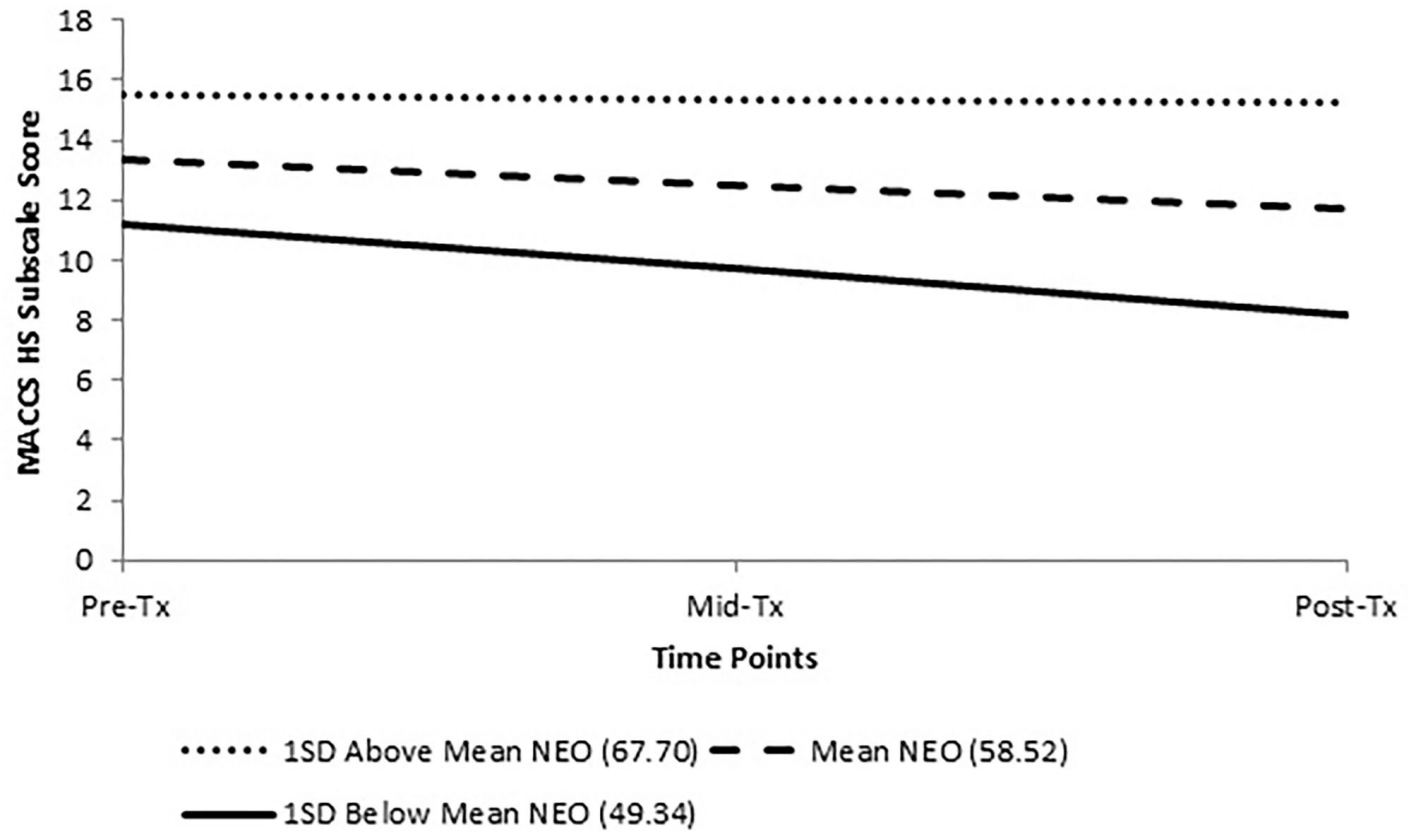
Change in MACCS High Standards Subscale score over treatment across levels of pre-treatment NEO Neuroticism Subscale (*T*-Score).

Full details of the two-level HLM analyses assessing the effect of baseline NEO Neuroticism Subscale *T*-Score on changes in MACCS over time can be found in Table 5. The only variable affected by baseline NEO Neuroticism was the MACCS High Standards Subscale. The addition of neuroticism to the model also pushed the change in High Standards over time to significance (*p* = 0.027, d = −0.45). This result is graphically represented in Figure 2, which indicates that those with higher baseline neuroticism have smaller decreases in MACCS High Standards over time relative to those with lower baseline neuroticism. Baseline HPS Self-Oriented Perfectionism did not have a significant effect on any of the MACCS subscales.

**Table 5:** Two-level hierarchical linear modelling assessing effect of baseline NEO Neuroticism *T* Score on change in MACCS total and subscale scores over time.

**Effect**	**b**	**SE**	**t**	**df**	***p***	**d**
**MACCS total score**						
Initial MACCS total (intercept)	94.51	2.37	39.95	25	<0.001	
NEO neurotic	1.14	0.26	4.37	25	<0.001	0.84
MACCS total over time (slope)	−2.97	0.60	−4.96	25	<0.001	−0.96
NEO neurotic	0.07	0.08	0.893	25	0.380	0.17
**MACCS attention/concentration subscale**						
Initial MACCS AC (intercept)	14.46	0.67	21.61	25	<0.001	
NEO neurotic	0.15	0.06	2.42	25	<0.001	0.47
MACCS AC over time (Slope)	−0.39	0.12	−3.25	25	<0.001	−0.63
NEO neurotic	0.02	0.01	1.51	25	0.159	0.29
**MACCS decision-making subscale**						
Initial MACCS DM (intercept)	16.75	0.75	22.24	25	<0.001	
NEO neurotic	0.26	0.06	4.17	25	<0.001	0.80
MACCS DM over time (slope)	−0.60	0.14	−4.33	25	<0.001	−0.83
NEO neurotic	0.01	0.02	0.32	25	0.753	0.06
**MACCS general memory subscale**						
Initial MACCS GM (intercept)	46.86	1.24	37.74	25	<0.001	
NEO neurotic	0.46	0.14	3.28	25	<0.001	0.63
MACCS GM over time (slope)	−1.66	0.33	−5.05	25	<0.001	−097
NEO neurotic	0.03	0.05	0.593	25	0.559	0.11
**MACCS High Standards Subscale**						
Initial MACCS HS (intercept)	13.35	0.74	17.95	25	<0.001	
NEO neurotic	0.23	0.06	3.83	25	<0.001	0.74
MACCS HS over time (slope)	−0.23	0.10	−2.35	25	0.027	−0.45
NEO neurotic	0.02	0.02	3.35	25	0.003	0.65

**Figure 3:**
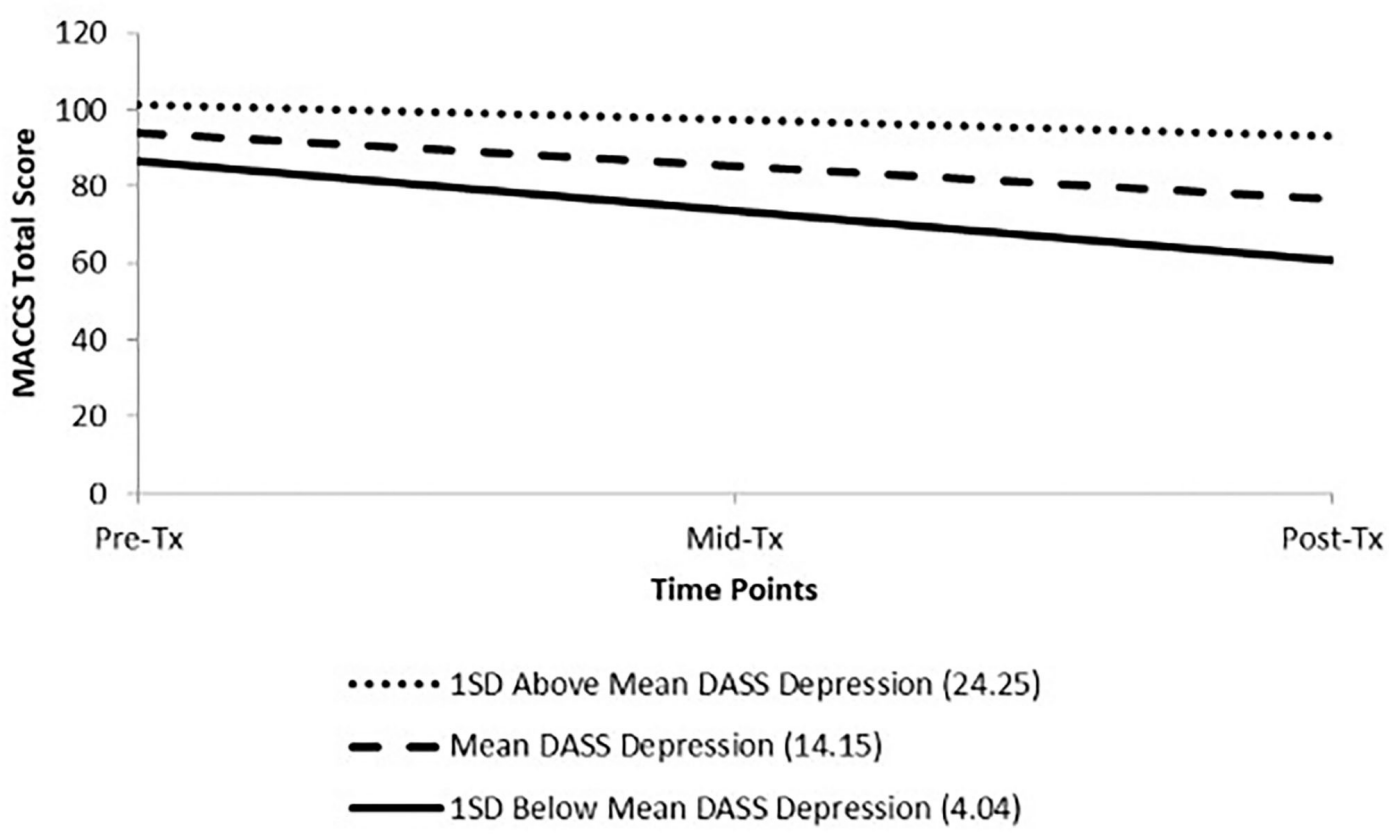
Change in MACCS Total Score over treatment across levels of pre-treatment DASS-21 Depression Subscale score.

Given the lack of significance on the DASS-21 subscales in the one-level models and observation of large standard deviations relative to the means (see Table 1), it was hypothesised *post-hoc* that varying levels of affective symptoms may also be contributing to variance in the change of MACCS scores over time. Table 6 shows the results of two-level HLM analyses assessing the effect of baseline DASS-21 subscales on change MACCS Total Score over time. The only significant Level-2 effect was that of baseline DASS-21 Depression (see Figure 3) indicating that those with higher depression scores at baseline had a smaller decrease in MACCS Total Score over time relative to those with lower ratings of depression.

**Table 6:** Two-level hierarchical linear modelling assessing effect of baseline on change in DASS-21 subscales on MACCS total score over time.

**Effect**	**b**	**SE**	**t**	**df**	***p***	**d**
**DASS-21 depression on MACCS total**						
Initial MACCS total (intercept)	94.03	2.83	33.22	24	<0.001	
DASS-D	0.74	0.25	2.95	24	0.007	0.58
MACCS total over time (slope)	−2.45	0.54	−4.56	24	<0.001	−0.89
DASS-D	0.12	0.05	2.33	24	0.029	0.46
**DASS-21 anxiety on MACCS total**						
Initial MACCS total (intercept)	93.99	3.15	29.81	24	<0.001	
DASS-A	0.18	0.45	0.39	24	0.699	0.08
MACCS total over time (slope)	−2.38	0.66	−3.63	24	0.001	−0.71
DASS-A	0.15	0.13	1.19	24	0.245	0.23
**DASS-21 stress on MACCS total**						
Initial MACCS total (intercept)	94.07	3.02	31.18	24	<0.001	
DASS-S	0.47	0.29	1.61	24	0.120	0.32
MACCS total over time (slope)	−2.57	0.61	−4.25	24	<0.001	−0.83
DASS-S	0.05	0.07	0.729	24	0.473	0.14

A *t*-test assessing the difference in neuroticism between those above and below the “moderate” cut-off (“moderate” range = 14–20) for DASS-21 Depression revealed that those at or above the cut-off (*n* = 14) had higher NEO neuroticism than those below the cut-off [*n* = 12; *t*_(25)_ = −3.21, *p* = 0.004]. Given this finding, we then performed a two-level HLM assessing the interaction effect of neuroticism and depressive symptoms on MACCS Total Score, as well as the MACCS High Standards subscale. Even though both DASS-21 Depression (*b* = 0.02, SE = 0.01, *t* = 3.04, *p* = 0.006) and NEO Neuroticism (see Table 4) were individually significant predictors of change in MACCS HS, the analyses including both predictors and the interaction effect were not significant; it likely that our small sample size precluded the sufficient power necessary to detect this interaction effect.

### One-Month Follow-Up

Data were collected for primary outcome variables at a 1-month follow-up appointment prior to the commencement of the booster session. A series of *t*-tests were performed to assess whether any gains observed in the primary analyses were maintained. Tests were performed for each of the MACCS subscales and total score, and none were statistically significant, indicating a maintenance of gains at 1-month follow-up, but no additional improvement.

### Satisfaction and Qualitative Feedback Data

Satisfaction and qualitative feedback were collected from 19 participants following group completion. Overall mean helpfulness was 4.95 (SD = 0.97) indicating overall the sample are reporting a high degree of helpfulness from participation in the group. Mean strategy use was 4.42 (SD = 0.84) indicating participants reporting regularly using the strategies they learned. When asked about co-occurring stressors during the group, seven women indicated there were no current stressors or left the section blank. Four women reported work stress, three had family and/or spousal discord/distress, two had personal medical issues, two others had bothersome depression and/or anxiety, while one person each indicated the following: empty nest, anniversary of interpersonal loss, moving houses, and family member health crisis. In terms of the duration of the group, nine participants indicated that the 5-session format was sufficient while six participants wanted additional sessions to explore the topics more in-depth or to focus on mastery of skills. Two participants thought the sessions should be longer in length to allow for more group discussion though one person thought a shorter session (e.g., 90 min) would suffice. Aside from two people who wanted additional content about topics beyond the scope of the group (e.g., urogenital concerns, relevant co-morbid medical conditions) and one who suggested the inclusion of mindfulness exercises, no participants had suggestions for the inclusion of topics that were not already covered in the program though several (*n* = 8) wanted greater depth of the material. Fourteen of the participants indicated that group discussion/support was among the most helpful aspects of the intervention while nine highlighted the psychoeducation followed by seven individuals who felt the specific strategies learned in the group were particularly beneficial. When asked about ways to improve the group, nine participants again indicated a preference for increased time spent on skill development and practise, with a preference for focusing on fewer goals and allotting more time to refinement and problem solving. One person suggested a break mid-way through each session, another indicated a need for reduced or free parking for group members, and another person requested a reading/resource list for additional menopause materials. Only two participants commented on group size; one thought the groups were too small while the other thought they were too large. Nine individuals had no feedback on potential improvements.

## Discussion

The purpose of this pilot study was to assess the feasibility of a 5-week cognitive remediation intervention to address women's subjective perception of cognitive difficulty and associated distress during the menopausal transition. Specifically, we hypothesised that the combination of psychoeducation, cognitive remediation strategies and development of lifestyle changes to promote brain health, presented in a group-based format would ameliorate subjective cognitive concerns and attenuate distress. Consistent with our hypothesis, all subscales of subjective cognitive confidence significantly improved over the course of the group (aside from high standards for one's cognitive abilities, which was trending in the significant direction). This suggests that overall, women's perception of, and confidence in, their cognitive abilities improved in the areas of attention/concentration, memory, and decision making. Gains made over the course of the group were maintained at 1-month follow-up. Interestingly, these gains were made in the absence of improvement in objective neuropsychological test performance or self-reported depression, anxiety, and stress. Neuropsychological test performance of our sample was, on average, within or above normal limits compared to same-aged peers, suggesting that women's self-reported cognitive abilities were perceived to be much lower than their actual performance. These findings align with other studies demonstrating no evidence for significant objective cognitive impairments during the menopausal transition (86). Only about 20% of the sample was taking HRT at the time of assessment so it is unlikely this entirely accounts for the strength of the neuropsychological data. These results are salient as approximately half of the sample had major depressive disorder which can impact cognitive functioning. While these findings may support the assertion that distress related to self-reported cognitive difficulties during menopause are, at least in part, mediated by psychological mechanisms, causality cannot be inferred at this time due to the lack of control condition. The finding that high standards toward one's cognitive abilities did not change as a function of group participation could reflect the small sample size of this study or average baseline perfectionism within the sample. Similarly, the intervention did not directly target mental health symptoms so a lack of change in mild to moderately elevated depression, stress, and anxiety is not entirely surprising.

Regarding feasibility, recruitment for this study was challenging despite using a variety of recruitment strategies. Most referrals came from an obstetrics and gynaecology clinic within our facility and it was unclear why other attempts to advertise this study more broadly were unsuccessful. As such, recruitment challenges are a limiting factor in conducting a larger-scale study. We had a 74% overall retention rate and most attrition was related to scheduling difficulties as many participants were still in the workforce, environmental factors, and/or personal issues unrelated to the group itself. This suggests that participants generally tolerated the intervention and that changing the timing of the intervention offered may improve attendance rates. Nonetheless, this intervention is relatively brief yet still produced significant results which makes it more practical to implement, particularly for women with multiple demands on their time. Self-reported helpfulness of the group and strategy use were high, indicating participants felt they benefitted from the intervention and were able to make changes in their lives (e.g., implementing strategies, good use of handouts and worksheets provided in the group). Qualitative feedback elicited from the post-intervention feedback form was very limited but focused on the therapeutic benefit of group discussion, appreciation of compensatory strategies, and value of the psychoeducation provided in the group that served to dispel myths about the menopause transition.

The secondary objective of this study was to gain a better understanding of factors or mechanisms involved in our intervention that promote clinical change. Subsequent analysis revealed more nuanced information regarding possible relationships between subjective cognitive concerns and psychological factors in response to the intervention. Higher baseline neuroticism was associated with less improvement in overall cognitive confidence compared to those with lower neuroticism while more severe baseline depression was associated with smaller decreases in high standards for one's cognitive performance following group completion. Contrary to our prediction, perfectionism did not have a significant impact on these variables, possibly because our sample reported average levels of perfectionism compared to same-aged peers in the community. Although this preliminary information may point to factors which could predict clinical outcomes in future groups, it should be noted that only about half our sample was in the clinical range for depression and even fewer in the clinical range for anxiety and stress. Although our results did not reveal an interaction effect between depressive symptoms and neuroticism on cognitive confidence, this may be a variable of interest in future studies with larger samples to ensure sufficient power.

### Limitations and Future Directions

A major limitation of this study is the lack of control condition which precludes casual interpretation of the data. For example, it is unclear if changes in cognitive confidence were due to the intervention itself or more general factors associated with group participation (e.g., increased socialisation and sharing of experiences). A replication study with the addition of a wait- control group could enhance methodology and potentially address these questions. The inclusion of a control group would also allow for an examination of learning (particularly rote verbal learning) with repeated assessment which has previously been identified as an area of cognition that could be impacted by menopause (15, 18, 87). Consideration could also be given to future test batteries to include potentially more ecologically valid tests of cognition such as the Rivermead Behavioural Memory Test—Extended version to assess subtle “every day” memory concerns that are reported during the menopause transition (88). Additionally, our participants tended to be affluent, well-educated, and somewhat homogenous in terms of ethnicity, and our sample size was quite small which limits generalizability of findings. A larger study is needed to confirm the results of these pilot data, ideally with a more diverse sample. Our study combined participants into one group rather than analysing cognitive data based on staging which may have revealed more variability in the data or differences between groups as previous studies suggest some variability in cognitive performance over the menopause transition (15, 17, 18).

In its current form, the Menopause and the Brain Group is strictly a cognitive remediation intervention consisting of psychoeducation, skill training, and group discussion without a psychotherapeutic component (e.g., cognitive behavioural therapy). However, providing the intervention within an acceptance and commitment-based framework (ACT) may provide therapeutic benefit as anecdotally many group discussions about implementing compensatory strategies revolved around accepting age-related changes, letting go of self-imposed standards based on prior levels of cognitive functioning, and values (e.g., creating time in one's schedule for self-care). Acceptance-based interventions have proved useful for other populations involving health and cognitive concerns and may have benefit when applied to women in the menopause transition (89–91).

In terms of possible mechanisms of change, it could be that group content improved participants' subjective sense of normalcy regarding subjective cognitive change and/or the emotional impact of perceived changes during the menopausal transition. Studies examining this construct have noted that when individuals do not understand their experiences (e.g., frequency of cognitive difficulties during menopause, normal age-related changes), they tend to believe their symptoms are unique and that they are less capable or competent than peers which can understandably translate into increased worry (92). Group interventions facilitate a normalisation process wherein individuals learn that others share their experiences and challenges, and improve adjustment difficulties (93). Indeed, qualitative information provided by study participants at the post-group assessment indicated a reduction in concerns about dementia following group completion for some, suggesting that their cognitive symptoms were normalised and contextualised. Other participants provided qualitative feedback reflecting a theme of normalising such as “It was beneficial learning that I'm not alone, that other women are experiencing the same things as me,” “that what I am experiencing is not in my head, there is biological evidence that explains what is happening,” and “other people are in the same boat.” One individual actually described the group as “normalising.” The concept of normalisation has been studied in normal ageing (94) which could be incorporated into future studies examining its potential role in the menopausal transition. Along these lines, qualitative impressions suggest that the formal inclusion of an individualised feedback session following group completion would be a useful addition to the treatment protocol and could augment the process of normalisation. Specifically, providing participants with information about objective cognitive performance and discussing subjective cognitive concerns within the context of menopausal and psychological factors could provide an impactful point of intervention and may enhance or solidify the information learned in the group.

## Conclusions

Preliminary data from our pilot study suggest a brief, 5-week cognitive intervention is well-tolerated and may improve cognitive confidence and reduce distress related to perceived cognitive decline during the menopausal transition. Further research is needed to confirm these findings and to identify possible mechanisms of change. This study contributes to the field by presenting results from a novel intervention addressing an underserved aspect of women's health during the menopause transition.

## Data Availability Statement

The raw data supporting the conclusions of this article will be made available by the authors, without undue reservation.

## Ethics Statement

The studies involving human participants were reviewed and approved by all research projects involvingMcMaster University, St. Joseph's Healthcare Hamilton and Hamilton Health Sciences physicians, staff (including staff acting as investigators outside the Institutions), students (i.e., research within the institution or using institutional resources), volunteers, visitors, or patients, must obtain ethical approval from the Hamilton Integrated Research Ethics Board (HiREB) before research can begin. This study received ethics approval from the HiREB on November 20, 2018 (reference number 2017-2950-GRA). All participants provided written informed consent. The patients/participants provided their written informed consent to participate in this study.

## Author Contributions

EB contributed to the design of the study, trained and supervised graduate students in data collection, co-wrote the grant and manuscript, co-facilitated the group, and coordinated the study. JK co-facilitated the group, contributed to the study design, and co-wrote the grant and manuscript. SG contributed to the study design and co-wrote the grant and manuscript. All authors contributed to the article and approved the submitted version.

## Funding

Funding for this study was provided by the Professional Advisory Committee (PAC) award from St. Joseph's Healthcare Hamilton, awarded March 26, 2018.

## Conflict of Interest

The authors declare that the research was conducted in the absence of any commercial or financial relationships that could be construed as a potential conflict of interest.

## Publisher's Note

All claims expressed in this article are solely those of the authors and do not necessarily represent those of their affiliated organizations, or those of the publisher, the editors and the reviewers. Any product that may be evaluated in this article, or claim that may be made by its manufacturer, is not guaranteed or endorsed by the publisher.
